# Supplementary material for: The technical-reasoning network is recruited when people observe others make or teach how to make tools: An fMRI study
Source: iScience. 2025 Jan 22;28(2):111870. doi: 10.1016/j.isci.2025.111870 (PMC11848787; doi:10.1016/j.isci.2025.111870)
Supplement: Document S1. Tables S1–S4 [file mmc1.pdf]

iScience, Volume *28*

Supplemental information

The technical-reasoning network is recruited when people observe others make or teach how to make tools: An fMRI study

Alexandre Bluet, Emanuelle Reynaud, Giovanni Federico, Chloé Bryche, Mathieu Lesourd, Arnaud Fournel, Franck Lamberton, Danielle Ibarrola, Yves Rossetti, and François Osiurak

This PDF file includes:

Tables S1 to S4

Table S1. Local maxima of activation clusters (MNI stereotactic coordinates) for the individual contrasts Teaching>Control, Observation>Control, Reverse engineering>Control and Teaching>Observation

Brain region	Hemisphere	Peak MIN			Cluster size	T-value	PFWE
		x	y	z			
Teaching>Control							
Postcentral Gyrus – PF[a]	Right	49	-18	38	909	16.13	< .001
Postcentral Gyrus – PF[a] – IPS[a]	Left	-34	-39	50	821	15.71	< .001
IOG	Right	30	-89	5	505	14.84	< .001
MOG	Left	-29	-92	-3	477	12.83	< .001
MTG – ITG[a]	Left	-45	-62	-1	453	11.22	< .001
ITG – MTG[a]	Right	46	-55	-12	284	11.15	< .001
IFG	Left	-47	7	27	104	9.80	< .001
Observation>Control							
Postcentral Gyrus – PF[a]	Right	51	-18	38	662	14.58	< .001
Postcentral Gyrus – PF[a] – IPS[a]	Left	-41	-30	43	484	11.98	< .001
IOG	Right	28	-89	-8	462	15.57	< .001
IOG	Left	-31	-89	-5	374	12.87	< .001
IOG – ITG[a]	Left	-43	-66	-3	155	9.26	< .001
Reverse engineering>Control							
IFG – MFG[a]	Right	46	16	36	455	11.40	< .001
ITG	Left	-43	-53	-8	197	12.20	< .001
Angular Gyrus	Right	33	-60	48	168	9.86	< .001
IPL	Left	-38	-46	41	133	8.76	< .001
ITG	Right	49	-48	-15	128	11.24	< .001
Cerebellum	Left	-11	-78	-35	119	10.24	< .001
IOG	Right	-31	-87	-8	114	13.45	< .001
Teaching>Observation							
Postcentral Gyrus	Right	28	-34	54	191	11.38	< .001
MTG	Right	44	-60	11	143	9.51	< .001

All results are thresholded at $p < .05$ (FWE, cluster level).

Brain region labels are given according the aal atlas.

[a] Regions that are part of our ROI but they are not the main peak in the cluster they are parts of.

IPS: inferior parietal sulcus; IOG: inferior occipital gyrus; MOG: middle occipital gyrus; MTG: middle temporal gyrus; ITG: inferior temporal gyrus; IFG: inferior frontal gyrus; MFG: middle frontal gyrus; IPL: inferior parietal lobule.

Table S2. Region activated in one-way repeated-measure ANOVA

Brain region	Hemisphere	Peak MIN			Cluster size	F-value	PFWE
		x	y	z			
Lingual Gyrus	Bilateral	10	-71	-5	2168	315.39	< .001
IOG – ITG[a] – MTG[a]	Right	28	-87	-8	2047	297.65	< .001
IOG – ITG[a]	Left	-31	-89	-5	2035	247.95	< .001
IPL – PF[a] – IPS[a]	Left	-50	-25	38	1357	200.83	< .001
IPL – PF[a]	Right	53	-21	38	1122	165.33	< .001
IFG – MFG[a]	Left	-50	9	27	374	106.77	< .001
Angular Gyrus	Left	-24	-76	34	343	78.20	< .001
IFG	Right	51	11	25	297	114.27	< .001
STG	Right	65	-39	15	288	68.14	< .001
Postcentral Gyrus	Right	5	-48	54	170	-	< .001
SPL	Right	26	-62	34	147	-	< .001
SMG	Left	-59	-41	22	146	-	< .001

All results are thresholded at $p < .05$ (FWE, cluster level).

Brain region labels are given according the aal atlas.

[a] Regions that are part of our ROI but they are not the main peak in the cluster they are parts of.

IOG: inferior occipital gyrus; ITG: inferior temporal gyrus; MTG: middle temporal gyrus; IPL: inferior parietal lobule; IPS: inferior parietal sulcus; IFG: inferior frontal gyrus; MFG: middle frontal gyrus; STG: superior temporal gyrus; SPL: superior parietal lobule; SMG: supramarginal gyrus.

Table S3. Post-hoc *t*-test results

Seed name	Teaching>Observation	Teaching>Reverse engineering	Observation>Reverse engineering
PF	.097[n.s.]	.000***	.020*
IFG	.096[n.s.]	.000***	.000***
IPS	.155[n.s.]	.002**	.074[n.s.]
left ITG	.016*	.000***	.018*
right ITG	.045*	.000***	.003**
MTG	.004**	.000***	.005**
right PF	.029*	.000***	.000***

n.s., not significant.
* $p < .05$
** $p < .01$
*** $p < .001$

Table S4. List of all tools being made in the video stimuli

Name	Description	Photo
Bucket	A safe bucket for children to carry toys.	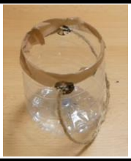
Container	A paper tray that can contain, for example, food.	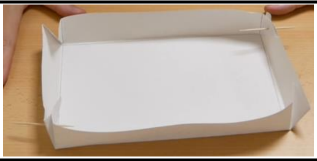
Dishrack	A dishrack for one plate.	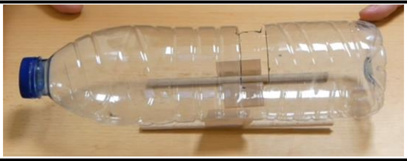
Kazoo	A small musical instrument that produces a buzzing sound.	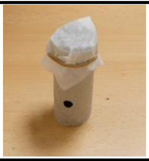
Rake	A small rake for a Zen garden.	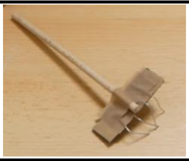
Roll holder	A roll holder for small roll that can be hung.	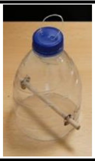
Seed starter	A seed starter with a water tank connected to a pot via a rope, which feeds the plant by capillarity.	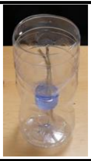
Shovel	A shovel for gardening.	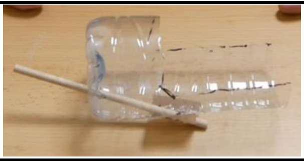
Slingshot	A slingshot that propels objects by pulling on its plastic part.	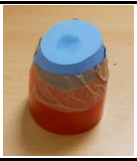
Transportable pot	A pot that can be closed and transported.	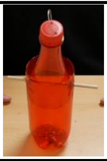
